# Effect of menopausal status on the survival and recurrence of sex-classified hepatocellular carcinoma after liver resection: a case-matched study with propensity score matching

**DOI:** 10.18632/aging.202155

**Published:** 2020-11-24

**Authors:** Wenli Zhang, Fuchen Liu, Jian Huang, Xinggang Guo, Wei Dong, Shuxun Wei, Li Li, Xiuli Zhu, Weiping Zhou, Hui Liu

**Affiliations:** 1 The Third Department of Hepatic Surgery, Eastern Hepatobiliary Surgery Hospital, Second Military Medical University, Shanghai 200438, China; 2 Changhai Hospital, Second Military Medical University, Shanghai 200438, China; 3 The First Department of General Surgery, Changzheng Hospital, Second Military Medical University and Naval Medical University, Shanghai 200438, China; 4 Department of Nephrology, Eastern Hepatobiliary Surgery Hospital, Second Military Medical University, Shanghai, China; 5 Department of Gastroenterology, Anhui Provincial Hospital, University of Science and Technology of China, Hefei, Anhui, China

**Keywords:** primary liver cancer, sex disparity, estrogen, prognosis, nomogram

## Abstract

Objective: To investigate the impact of menopausal status on the prognosis for sex-classified Hepatocellular carcinoma (HCC) and to establish prognostic nomograms for patients after liver resection.

Results: After propensity score matching (PSM), statistically significant differences in both overall survival (OS) and recurrence-free survival (RFS) were found between men and women HCC patients. Based on Cox regression analysis, these differences were evident in the normal menstruation (N) group expanded with male patients, but not in either the expanded postmenopausal (P) or intermediate (I) groups. Sex disparity was also apparent in the recurrence-free survival (RFS) of the total HCC patients. Integrated with independent factors, nomograms for the OS and RFS of the expanded N group showed higher C-indices of 0.773 and 0.724, respectively, than those of nomograms for the total patients and BCLC stage (P<0.001).

Conclusion: Sex disparity appears to affect both the survival and recurrence of HCC only in normal menstruation women and their matched men. For predicting survival, prognostic nomograms derived from the expanded N group of HCC patients were more accurate for patients with the same clinical conditions.

Methods: The patients (390 females and 1920 males), who underwent curative liver resection for HCC during 2008 to 2012, were screened. The 390 women were divided into three groups: normal menstruation, intermediate, and postmenopausal. To overcome selection bias, the three groups of females were matched with males at a ratio of 1:2, using propensity score matching. Based on further Cox regression analysis, independent factors were integrated into nomograms for OS and RFS by R rms. The accuracy and discrimination of the nomograms were evaluated by the C-index, calibration curve, and decision curve analysis.

## INTRODUCTION

Hepatocellular carcinoma (HCC), which ranks as the sixth most common cancer and the third-highest cause of cancer deaths worldwide, is affected by various etiological factors, including hepatitis B or hepatitis C virus infection, tobacco, or alcohol overconsumption, and metabolic syndrome [1]. Among these factors, chronic hepatitis B virus (HBV) exposure contributes to 50 percent of total HCC cases while the impact of sex is being progressively uncovered [2, 3]. According to recently updated cancer statistics and cohort research, females have a lower incidence and mortality but the studies around survival are limited in number and the data is inconsistent [4, 5].

To investigate the distinct male predominance of HCC, several researchers focused on the potential role of sex hormones and identified critical and opposite roles played by these hormones in tumor cell proliferation and the immune response to viral infection [6]. It was proposed that estrogen had a protective effect leading to a reduced susceptibility to viral infection and a stronger immune response, while high androgen levels tended to promote carcinogenesis [7–11]. Although experimental castration models with administration of estrogen or antiandrogen suppressed HCC development, clinical trials of antiandrogen treatment to HCC did not show improved survival [12, 13]. Given that no definite conclusion was drawn from the sex hormone studies, and the controversy around the sex disparity in HCC and HCC-related risk factors, additional studies with larger numbers of female patients should focus on more detailed aspects in view of the complexity of HCC.

Liver resection is still being evaluated as the predominant therapeutic option for compensated cirrhosis patients [1]. Although different levels of sex hormones, immune responses, and obesity have been noted in sex-based studies on the risk of HCC, few studies have investigated the influence of menstruation status on the prognosis of patients with surgical resection as primary treatment [14, 15].

To determine the sex disparity and the influence of menstruation status on HCC prognosis, a population of HCC patients undergoing radical resection were enrolled in the present study. Here, we report our verification of the association between menstruation status and survival using a propensity score matching (PSM) approach to avoid selection bias. We also present predictive nomograms for the different patient groupings.

## RESULTS

### Descriptive data and grouping based on propensity score matching

**Figure 1:**
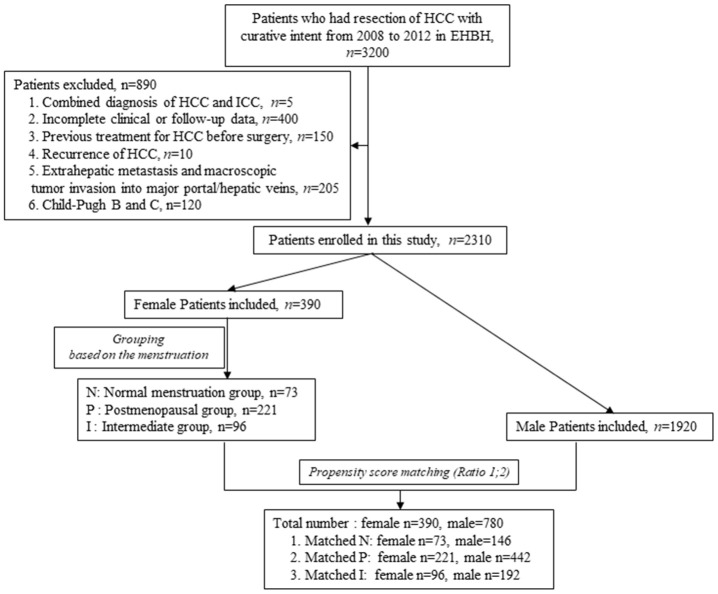
**Flow chart of HCC patients screening and grouping.**

According to the inclusion criteria described in the methods, a total of 2310 HCC patients were enrolled in this study, including 390 female and 1920 male patients. According to the propensity score matching method, the female patients of three groups were matched with the pool of 1920 male patients at a ratio of 1:2, respectively (Figure 1) to balance the selection bias of male and female HCC patients. After propensity score matching, a total of 1170 HCC patients, including 390 female and 780 male cases, were enrolled in the study for further analysis. The details of each pair of the matched groups are shown in Figure 1.

### Sex disparity in the prognosis of total patients with HCC

**Figure 2:**
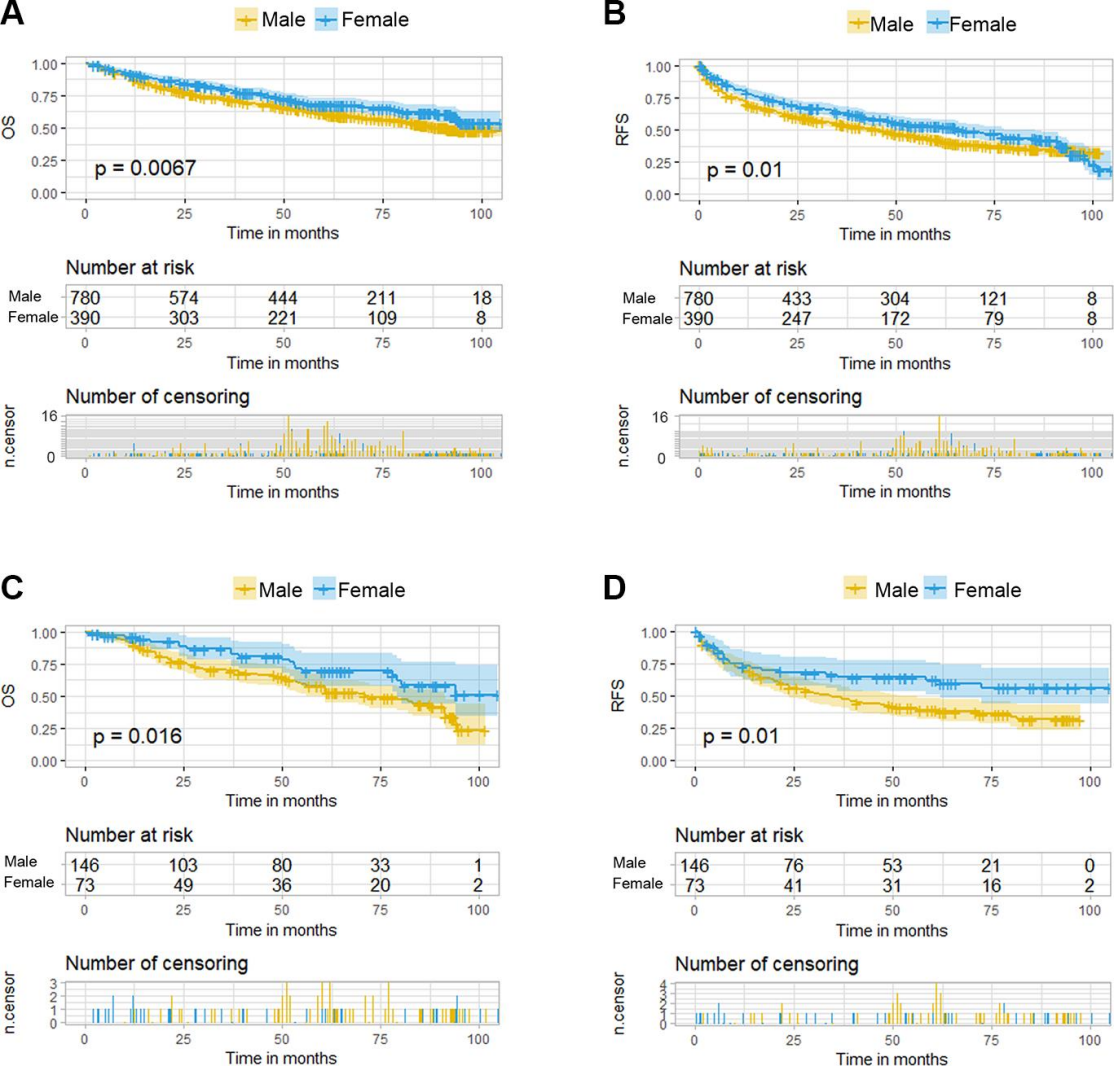
**Kaplan-Meier curves for overall survival (OS) and recurrence free survival (RFS) of HCC patients grouped by sex. (A**, **B**). KM curves of OS and RFS in total HCC patients. (**C**, **D**) KM curves of OS and RFS in the normal menstruation females and their matched males.

HBV infection was prevalent among the 1170 patients and could be classified as high- or low-level infection based on virus titration. The distribution of demographic, laboratory, and clinical features were not significantly different between the male and female patients, with the exception of cigarette smoking, alcohol consumption, diabetes mellitus, γ-glutamyl transferase (GGT) level, and microvascular invasion (MVI) (Supplementary Table 1). Based on the Kaplan-Meier survival curves, significant differences between the female and male HCC patients existed in either overall survival (OS) or recurrence-free survival (RFS) (Figure 2A and 2B). Furthermore, uni- and multivariate Cox regression analyses were conducted to explore the survival difference between female and male HCC patients, and the results showed that sex was an independent factor only for RFS of HCC patients (Supplementary Table 2), which was partly consistent with the previous study. The previous study showed that while men had a significantly higher risk of late recurrence (>2 years) and rates of cancer-specific mortality after HCC resection than women, there was no significant difference in overall survival, which is consistent with the analysis in our study [16].

### The survival of postmenopausal HCC female patients and their matched male patients were not significantly different

In the expanded P group, only nine features among the 24 enrolled factors were significantly different between preoperative postmenopausal women and their matched men with HCC (Supplementary Table 3). The KM curves of OS and RFS for female and male patients were compiled and no statistical difference was found in survival (Supplementary Figure 1A and 1B). The Cox regression model was used to analyze OS and RFS. The univariate Cox-regression analysis of the P group showed that Alb, AST, GGT, ALP, tumor size, number of tumors, satellite nodules, cirrhosis, Edmondson-Steiner grade staging, and MVI are the common factors for OS and RFS. Tbi and α-fetoprotein (AFP) were risk factors only for OS while ALT and HBV_DNA levels were only risk factors for RFS. After further multivariate Cox regression analysis, six and four independent risk factors were identified for OS and RFS, respectively. Notably, sex difference was not among them (Supplementary Table 4).

### There was no significant difference between the survival of intermediate group females and their matched male patients

In the I group expanded with male patients, the basic features of the HCC patients were well balanced between the sexes (Supplementary Table 5). The Kaplan-Meier survival curves of OS and RFS between the two sexes also showed no statistical difference (Supplementary Figure 1C and 1D). The Cox regression analysis revealed that GGT, Edmondson-Steiner grade, and tumor size were independent risk factors for OS while AST and tumor size were independent risk factors for RFS (Supplementary Table 6). These results further demonstrated that sex was not an independent factor for survival in the I group.

### The survival of female patients with normal menstruation was better than their matched male patients

Under the propensity score matching, the matched N group patients showed good balance between the sexes. There was no significant difference in the distribution of demographic, laboratory, and clinical features except for cigarette smoking, alcohol consumption, antiviral treatment and Edmondson-Steiner grade staging (Table 1). The OS was better in female patients than that in the matched male patients (p=0.016, Figure 2C) and this significant advantage was also present in the RFS (p=0.01, Figure 2D). Multivariate Cox regression analysis among these two groups demonstrated sex difference as an independent risk factor for both OS and RFS (Table 2). Besides the sex disparity, five and four factors were additional independent risk factors for OS and RFS of HCC patients, respectively.

**Table 1:** Comparison of clinicopathological characteristics between normal menstruation females and their matched male HCC patients after PSM.

**Features**	**Male (n=146)**	**Female (n=73)**	***P*-value**
Age (mean,Years)	38.49 (4.70)	37.49(5.74)	0.173
Cigarette smoking (Yes/No)(%)	75(51.4)/71(48.6)	2(2.7)/7(97.3)	**<0.001**
Alcohol consumption (Yes/No)(%)	83(56.8)/63(43.2)	3(4.1)/70 (95.9)	**<0.001**
Diabetes mellitus (Yes/No)(%)	6(4.1)/140(95.9)	3(4.1)/70(95.9)	0.189
Antiviral (Yes/No)(%)	39(26.7)/107(73.3)	9(12.3)/64 (87.7)	**0.024**
Tbi(mean, μmol/L)	12.48(10.03)	11.75 (4.75)	0.554
Alb(mean, g/L)	42.09 (3.69)	42.41 (4.11)	0.564
ALT(mean, U/L)	36.23 (33.15)	32.89(49.31)	0.552
AST(mean, U/L)	36.06 (27.29)	42.21(50.67)	0.245
GGT(mean, U/L)	72.87 (67.73)	58.53(79.00)	0.164
ALP(mean, U/L)	87.53 (45.08)	90.81 (74.88)	0.688
AFP(mean, ng/ml)	664.48 (2230.08)	675.90 (549.97)	0.966
HBsAg(Positive/Negative)(%)	144(98.6)/2(1.4)	68 (93.2)/5(6.8)	0.077
HBVDNA(≥2000/<2000 IU/ml)(%)	73(50.0)/73(50.0)	35(47.9)/38 (52.1)	0.886
Type of resection (Nonanatomical/anatomical)(%)	73 (50.0) /73(50.0)	33(45.2)/40(54.8)	0.599
Surgical margin (<1/≥1cm)(%)	60(41.1)/86(58.9)	32(43.8)/41(56.2)	0.809
Hilar clamping time (mean, minites)	15.84(8.08)	16.96 (12.41)	0.425
Tumor size(mean, cm)	5.24(3.77)	6.16 (4.83)	0.126
Tumor Number (>1/1)(%)	13(8.9)/133(91.1)	6(8.2)/67(91.8)	1.000
Satellite nodules (presence/absence)(%)	31(21.2)/115(78.8)	25(34.2)/48(65.8)	0.055
Cirrhosis(Yes/No)(%)	101(69.2)/45(30.8)	42(57.5)/31(42.5)	0.120
Edmondson-Steiner grade III-IV/I-II(%)	103(70.5)/43(29.5)	38(52.1)/35(47.9)	**0.011**
MVI (Yes/No)(%)	33(22.6)/113(77.4)	26(35.6)/47(64.4)	0.059
BCLC stage (A-B/0)(%)	135(92.5)/11(7.5)	71(97.3)/2(2.7)	0.266

Bold values indicate statistical significance (*P* < 0.05). *P* values male versus female cohort.

HCC, hepatocellular carcinoma; Tbi, total bilirubin; Alb, albumin; ALT, alanine transaminase; AST, aspartate aminotransferase; GGT, γ-glutamyl transferase; ALP, alkaline phosphatase; AFP indicates α-fetoprotein; MVI, microvascular invasion; BCLC, Barcelona Clinic Liver Cancer.

**Table 2:** Univariate and multivariate analysis of OS and RFS in the normal menstruation group matched with males.

**Features**	**OS**			**RFS**		
	**HR**	**95%CI**	***P*-value**		**HR**	**95%CI**	***P*-value**
**Univariate analysis**							
Sex(Male/Female)	0.550	0.33-0.9	**0.017**		0.570	0.37-0.88	**0.011**
Age (mean,Years)	0.990	0.95-1.03	0.491		0.980	0.95-1.02	0.353
Cigarette smoking (Yes/No)(%)	1.170	0.77-1.76	0.465		1.040	0.71-1.52	0.856
Alcohol consumption (Yes/No)(%)	1.320	0.88-1.99	0.181		1.300	0.9-1.87	0.167
Diabetes mellitus (Yes/No)(%)	1.040	0.38-2.83	0.946		2.060	1-4.23	0.050
Antiviral (Yes/No)(%)	1.110	0.7-1.77	0.656		0.750	0.47-1.2	0.233
Tbi(mean, μmol/L)	0.970	0.93-1.01	0.170		0.980	0.95-1.01	0.226
Alb(mean, g/L)	0.960	0.91-1.01	0.141		0.970	0.92-1.01	0.157
ALT(mean, U/L)	1.000	0.99-1.01	0.452		1.000	0.99-1	0.453
AST(mean, U/L)	1.010	1-1.01	**0.006**		1.000	1-1.01	0.320
GGT(mean, U/L)	1.000	1-1.01	**<0.001**		1.000	1-1.00	**0.002**
ALP(mean, U/L)	1.000	1-1.01	**0.007**		1.000	1-1.01	**0.001**
AFP(mean, ng/ml)	1.000	1-1.00	0.177		1.000	1-1.00	0.625
HBsAg(Positive/Negative)(%)	1.590	0.39-6.47	0.516		4.310	0.6-30.89	0.146
HBV_DNA(≥2000/<2000 IU/ml)(%)	1.680	1.11-2.54	**0.014**		1.430	0.99-2.07	0.058
Type of resection (Nonanatomical/anatomical)(%)	0.970	0.65-1.46	0.894		0.980	0.68-1.41	0.907
Surgical margin (<1/≥1cm)(%)	1.990	1.32-2.99	**0.001**		1.230	0.85-1.78	0.270
Hilar clamping time (mean, minites)	1.000	0.98-1.03	0.857		1.010	0.99-1.04	0.215
Tumor size(mean, cm)	1.110	1.06-1.15	**<0.001**		1.080	1.04-1.12	**<0.001**
Tumor Number (>1/1)(%)	0.630	0.29-1.37	0.241		1.630	0.92-2.91	0.096
Satellite nodules (presence/absence)(%)	2.260	1.44-3.55	**<0.001**		2.960	1.99-4.42	**<0.001**
Cirrhosis(Yes/No)(%)	1.180	0.76-1.83	0.461		1.170	0.79-1.74	0.431
Edmondson-Steiner grade III-IV/I-II(%)	2.090	1.34-3.25	**0.001**		1.960	1.31-2.93	**0.001**
MVI (Yes/No)(%)	2.610	1.69-4.06	**<0.001**		2.320	1.56-3.44	**<0.001**
**Multivariate analysis**							
Sex(Male/Female)	0.530	0.31-0.9	**0.019**		0.530	0.34-0.85	**0.008**
HBV_DNA(≥2000/<2000 IU/ml)(%)	1.460	0.94-2.25	0.090				
Surgical margin (<1/≥1cm)(%)	2.210	1.42-3.44	**<0.001**				
Tumor size(mean, cm)	1.150	1.08-1.22	**<0.001**		1.060	1-1.12	**0.033**
Satellite nodules (presence/absence)(%)	2.060	1.26-3.39	**0.004**		2.710	1.79-4.11	**<0.001**
Edmondson-Steiner grade III-IV/I-II(%)	1.770	1.09-2.89	**0.022**		1.620	1.06-2.49	**0.027**
MVI (Yes/No)(%)	2.320	1.38-3.92	**0.002**		1.950	1.27-2.99	**0.002**

Bold values indicate statistical significance (*P* < 0.05).

HCC, hepatocellular carcinoma; Tbi, total bilirubin; Alb, albumin; ALT, alanine transaminase; AST, aspartate aminotransferase; GGT, γ-glutamyl transferase; ALP, alkaline phosphatase; AFP indicates α-fetoprotein; MVI, microvascular invasion; BCLC, Barcelona Clinic Liver Cancer; OS, overall survival; RFS, recurrence-free survival; CI, confidence interval; HR, hazard ratio.

To summarize, the RFS and OS of HCC between female patients and their matched male patients showed obvious differences in the normal menstruation group, which were not evident in the postmenopausal and intermediate groups.

### Modeling the risk for predicting prognosis of normally menstruating females and their matched male HCC patients and accessing its evaluation by the calibration curve

**Figure 3:**
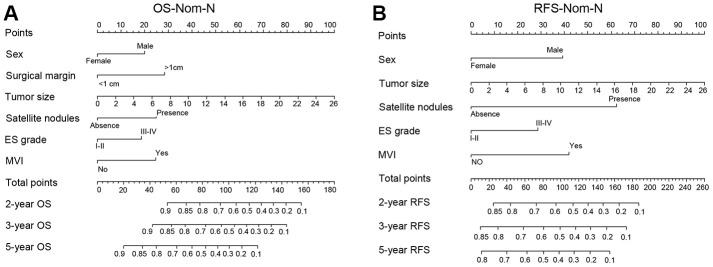
**Nomograms for predicting prognosis of the matched normal menstruation group patients with HCC after PSM.** (**A**) nomogram model of Overall survival (OS) (OS-Nom-N). (**B**) nomogram model of Recurrence free survival (RFS) (RFS-Nom-N).

**Figure 4:**
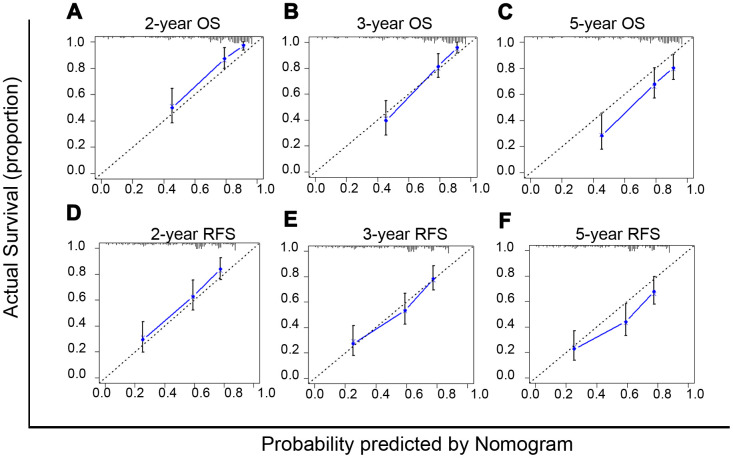
****Calibration curves of nomograms for predicting overall survival (OS) (**A**–**C**) and recurrence free survival (RFS) (**D**–**F**) at 2-, 3- and 5-year in the matched normal menstruation group patients with HCC after PSM.

The nomogram model can predict outcomes and provide a risk evaluation for each patient based on screened independent risk factors. This has been shown to be more accurate in predicting prognosis than other conventional staging systems. In the N group matched with men, the above independent factors for OS and RFS were integrated into nomogram models (OS nomogram of matched N group named OS-Nom-N, RFS nomogram of matched N group named RFS-Nom-N), respectively (Figure 3A, 3B). The C-index and calibration curve were used to evaluate the consistency between the predictions based on the nomogram models and the actual value. The models performed well for predicting OS and RFS. The C-index of OS-Nom-N and RFS-Nom-N models were 0.773 (95%CI, 0.726-0.820) for OS and 0.724 (95%, 0.676-0.771) for RFS (Table 3). The calibration curves (2-, 3-, or 5-year) showed a reasonable uniformity between the prediction by nomogram models (OS-Nom-N and RFS-Nom-N) and the actual observation (Figure 4A–4F).

**Table 3:** C-index of prognostic staging systems for OS and RFS in patients with HCC.

**Staging Systems**	**OS**				**Staging Systems**	**RFS**		
	**C-index**	**95%CI**	***^$^P***			**C-index**	**95%CI**	***^&^P***
OS-Nom-N	0.773	0.726-0.820			RFS-Nom-N	0.724	0.676-0.771	
OS-Nom-T	0.718	0.695-0.741	<0.001		RFS-Nom-T	0.659	0.637-0.680	<0.001
BCLC stage	0.513	0.509-0.516	<0.001		BCLC stage	0.523	0.505-0.538	<0.001

^$^*P* values OS-Nom-N versus other stage systems. ^&^*P* values RFS-Nom-N versus other stage systems.

BCLC indicates Barcelona Clinic Liver Cancer; CI, confidence interval; C-index, concordance index; HCC, hepatocellular carcinoma; OS, overall survival; RFS, recurrence-free survival.

### Performance of the nomogram models for the normal menstruation group matched with male HCC patients

Based on the above results, sex disparity was an independent risk factor for OS only in females with normal menstruation and their matched male HCC patients. However, for RFS, sex was an independent factor not only in N group patients matched with men but also in the total HCC patients. Then, according to the above Cox-regression analysis (Supplementary Table 2), the nomogram models for the OS and RFS of the total HCC patients were developed (OS nomogram of total HCC patients named OS-Nom-T, RFS nomogram of total HCC patients named RFS-Nom-T) (Supplementary Figure 2). BCLC (Barcelona Clinical Liver Cancer) is the most common staging system in clinical practice for HCC. To define a more reasonable and efficient model for predicting survival, the above three models (Nom-N, Nom-T, and the BCLC model) were compared based on the C-index which is proportional to the predictive power.

For HCC OS prediction, the indices of the OS-Nom-T and BCLC were 0.718 (95%CI, 0.695-0.741) and 0.513 (95%CI, 0.509-0.516), respectively, both of which were significantly lower than that of the OS-Nom-N model (C-index 0.773 with 95%CI, 0.726-0.820; *P*<0.001, *P*<0.001) (Table 3). For RFS prediction, the index of the RFS-Nom-N was higher than that of both RFS-Nom-T and BCLC stage (*P*<0.001, *P*<0.001) (Table 3). Therefore, with its powerful prediction ability, the OS-Nom-N model was deemed more accurate and reliable than the other two models for survival prediction.

### Evaluation of the clinical utility of the nomogram models by DCA

**Figure 5:**
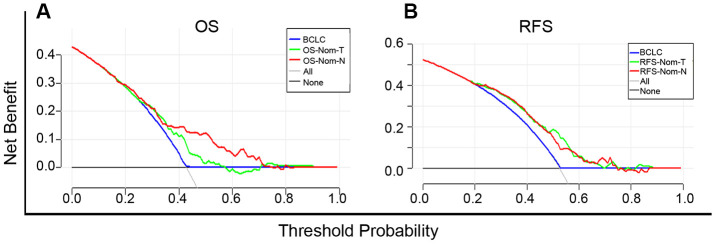
Decision curve analysis (DCA) curves for overall survival (OS) (**A**) and recurrence-free survival (RFS) (**B**) of established prognostic models. The x-axis and the y-axis represent the threshold probability and net benefit, respectively. Solid black line (None): no patients will experience the event. Solid gray line (All): all patients will die.

Based on the net benefit derived from a range of threshold probabilities, decision curve analysis (DCA) curves were constructed for evaluating the ability of the prediction models and, therefore, to direct clinical practice. The clinical application was assessed by comparison with the BCLC stage and nomogram models according to the DCA curves. For predicting OS, in a wide range of threshold probabilities, OS-Nom-N performed better than OS-Nom-T and BCLC stage (Figure 5A). For predicting RFS, RFS-Nom-N showed a similar net benefit with RFS-Nom-T across a wide range of threshold probabilities, and both were better than BCLC (Figure 5B).

## DISCUSSION

In our current study, 390 female patients and 1920 male HCC patients treated with curative resection as the initial therapy were included. There were between-sex differences for the OS and RFS of HCC in the normal menstruation subgroup but not in the intermediate or postmenopausal subgroups. Thus, we developed and validated nomograms based on Cox regression analysis to predict the 2-, 3- and 5-year survival for all HCC patients, restricting our procedure to normally menstruating females and their matched male patients for more accurate prediction.

Comparison of our nomograms with the BCLC staging system illustrated that Nom-N was more powerful than Nom-T and BCLC for OS and RFS prediction in terms of the C-index. The net clinical benefit of OS-Nom-N was evident in DCA where the threshold probability ranged between 40% and 70% while obvious superiority was seen in both RFS-Nom-N and RFS-Nom-T when compared to BCLC.

A higher incidence of HCC in men than in women has been observed in a large number of previous studies; however, studies on the effect of sex difference on survival are limited and controversial. Consistent with our present study, some researchers found that female HCC patients had better survival than male patients while others considered that female sex was not an independent factor or that there were survival differences among patients of all ethnicities except Asian [3, 5, 17]. In contrast to previous studies that enrolled patients regardless of race and treatment, we conducted the current research in patients of uniform race and who had initially undergone surgical treatment only. Furthermore, different strategies of grouping matching were used in our research.

The majority of related studies conducted analyses with subjects stratified by age [18]. Notably, menstruation status is closely related to but not equivalent to age, which could partially mask the effect of the former. Menopause usually occurred at age 51 with a 10% exception between ages 40-45 or after 55 [19]. As clear menstruation status was able to be extracted from the patients’ history, we grouped the subjects by menstruation for further analysis. Propensity score matching has been proposed as a method for overcoming selection bias and increasing the credibility of observational studies [20]. Hence, this method was used to match our selected female patients with suitable male patients. In the three subgroups, factors were optimally balanced when comparing the survival.

In accordance with previous studies, the protective effect of female sex was found to gradually decrease with increasing age [3]. Some reports have suggested that estrogen plays a crucial rule in the protective effect [6, 10]. The liver is considered to be a sexually dimorphic organ with expression of both estrogen and androgen receptors [21]. Different levels of sex hormones affect the genetic pattern and immune response to exotic substances [22, 23]. In addition, a close relationship has been found between the level of estrogen or androgen and the risk of HCC [24]. Oral contraceptive drugs can not only induce benign hepatoma but can also accelerate the progression of HCC in animals [25–27]. Conversely, evidence has shown that estrogen replacement can prolong the survival time of female HCC patients and reduce the risk of liver cancer [28, 29]. As for androgen, elevated testosterone levels have been shown to promote HCC in cirrhosis patients while two clinical trials failed to confirm the effectiveness of antiandrogen treatment [13, 30, 31]. Moreover, the assumption that estrogen level was the main factor affected by menopause ignored the conversion from androgen to estrogen carried out by aromatase, which can also be influenced by menopause [32]. However, the action of sex hormones and their receptors in HBV infection and the immune response to it could be the most likely explanation for the observed sex-related menopausal status-influenced HCC prognosis. It has been reported that in both acute and persistent HBV infection female mice had higher CD8^+^ T cell activity and lower intrahepatic Treg cells than male mice [33]. In addition to innate immune cells, cytokines, such as IL-6, can be transcriptionally inhibited by estrogen from Kupffer cells via the reduction of NF-κB [34]. In contrast, androgen can upregulate the viral antigen titer as shown by higher levels of HBsAg in male mice when compared to female ones and a reduction after castration [35, 36]. Mechanistically, androgen and its receptor could bind to two androgen-responsive element motifs within the genomic region of the HBV enhancer I to increase the HBV titer and enhance the transcription of TERT, whose activation might be essential for HCC development from cirrhosis, after HBV integration in the TERT promoter, while estrogen and its receptor had inverse actions [7, 24, 37]. As the level of estrogen in females declines after menopause and hormones in males are quite stable up to 60-years-old, it is reasonable to consider sex hormones as the main reason for the different prognosis between two sexes. However, the exact mechanism of sex hormone action on HCC recurrence warrants further investigation. In addition, sex differences can also be found in some identified HCC risk factors. For example, Xie et al reported distinct sex differences in gut microbiota, bile acids, and microRNA profiles, which could contribute to liver carcinogenesis [38]. Moreover, there were sex-based disparities in the expression of inflammatory cytokines and compliance to screening or surveillance [17].

Some research has shown that excessive alcohol consumption is negatively related to HCC prognosis [39–42]. However, we could not draw the conclusion either from all HCC patients (Supplementary Table 2) or the three subgroups (Table 2, Supplementary Tables 6, 4). There might be several reasons for the phenomenon. Firstly, the subjects of the research cited above included patients purely on the basis of alcohol consumption and excluded patients with mixed causes of liver disease. In contrast, while most of the patients in our cohort had HBV infection, only 31.3% of males and 2.1% of females had associated alcohol-related HCC etiology [39]. Secondly, it is well known that females have lower alcohol consumption rates compared to males [43]. We selected our male patients under the PSM method, which could lead to a set that does not reflect the true drinking status in men. Thirdly, it has been reported that alcoholic HCC was detected outside any surveillance much more frequently [44] and at a more advanced stage than HCV HCC [40], even though the HCC stage contributed more than the alcohol etiology itself to poorer prognosis. Dunbar et al believed that alcohol consumption might influence survival by delaying patients from seeking treatment and encouraging poor adherence to treatment [41]. If a patient has mixed etiologies with HBV and alcohol, HBV treatment might assist with the surveillance. Nevertheless, only BCLC A and B patients who could potentially be cured by surgical resection were included in our study according to the inclusion criteria. Thus, it might be difficult to observe significance. Furthermore, Raffetti et al retrospectively collected alcoholic HCC patients undergoing various treatments and found no higher risk in hepatitis virus + alcohol than in hepatitis virus alone [42]. Lastly, our data is not suitable for the analysis of alcohol consumption with HCC prognosis. The clinical pattern of alcohol-related liver disease shows frequent association with diabetes and overweight/obesity, multi-nodularity and low production of AFP. These patients are in a minority in our data, which could reflect a distinct carcinogenetic pattern. Also, there are differences between Asians and other races.

Basing on our new nomogram for RFS, the 2-year RFS prediction for a 35-year-old female with low HBV titration, two-centimeter diameter tumor, no satellite nodules and MVI, no cirrhosis and ES grade I is higher than 0.85 while the 2-year RFS for a male patient in the same condition was below 0.8. However, in RFS-Nom-T, the predicted probability of recurrence for these two patients are all around 0.85. The nomogram is a simple, visual, and commonly used method for probability prediction. The BCLC staging system is the most common clinical staging system for HCC patients for evaluating their condition, treatment decisions, and prognosis prediction. However, our nomograms exhibited higher net benefit in both C-index and DCA curves for both OS and RFS, which may guide follow-up frequency.

There are also limitations in the present study. For example, this was a retrospective study and did not collect blood samples from patients, we could not specifically of estrogen concentrations before and after menopause. Also, sex disparity-related etiological factors were not included in further analysis. Whether sex hormones are the single direct influencing factor or other factors, such as circadian rhythms or epigenetic alterations, that may influence and be influenced by sex hormones in complex ways are also involved warrants more research.

In conclusion, the nomograms in our study depicted the OS and RFS more accurately than the present predictive guidelines. Based on propensity score matching, we performed balanced subgroup analyses in three female subgroups defined by menstruation status and their matched male counterparts to achieve precise prediction and provide evidence for the usefulness of menopause hormone therapy (MHT) for postmenopausal women. It might also support more frequent follow-ups in male patients matched with females in the normal menstruation group for early diagnosis and treatment.

## MATERIALS AND METHODS

### Patients

From 2008 to 2012, a total of 3200 patients who had been diagnosed with HCC and had undergone surgical resection as the primary treatment in Eastern Hepatobiliary Surgery Hospital, Second Military Medical University, were screened and enrolled in our analysis. The early diagnosis of HCC was based on typical imaging features on dynamic CT and/or MRI and the level of AFP in accordance with current guidelines, and was further confirmed by postoperative pathological diagnosis. We identified 390 female and 780 male HCC patients who met the inclusion criteria: (1) a clear and definite record of menstruation at the time of surgery and during the follow-up for female patients, (2) Child-Pugh score A, (3) no previous treatment for HCC before surgery, (4) radical hepatectomy (R0), (5) HCC the only diagnosis of malignancy, (6) no extrahepatic metastasis and macroscopic tumor invasion into the major portal/hepatic veins and bile duct (Figure 1). A female patient who had had regular menstruation until the end of the follow-up was assigned to the normal menstruation group (N) while a patient who had had menopause before surgery was assigned to the postmenopausal group (P). The remaining patients whose menopause occurred during the follow-up were included in the intermediate group (I) (Figure 1).

### Clinicopathological characteristics and definitions

Demographic characteristics, surgical information, and results of laboratory tests were recorded in detail. For demographic characteristics, we collected information about age, sex, menstruation, diabetes mellitus, smoking, and alcohol consumption. The laboratory tests mainly included tests for liver function and virus infection and were carried out before surgery. Surgical information covered the pathological examination of the tumor and recorded resection process. Details are listed in Supplementary Table 1.

Our definition of alcohol consumption corresponds to alcohol abuse (i.e. alcohol consumption over five years: ethanol≥40g/d for males,≥20g/d for females, or a history of heavy drinking within a two week period: ethanol≥80g/d) [45]. The tumor differentiation grade was scored based on the Edmondson-Steiner classification [46]. Malignant emboli were found in hepatic and portal veins and lymphatic ducts under microscopy, which was defined as microvascular invasion (MVI). Definitions of satellite nodules and BCLC stage are described in our previous study [47]. Complete excision of the tumor-burdened portal tributaries was defined as anatomical resection [48] with its opposite being non-anatomical resection. The use of the clinical data for research was communicated to the patients and informed consent forms were signed with each patient. The study was approved by the Ethics Committee of Eastern Hepatobiliary Surgery Hospital.

### Follow-up

The follow-up procedure was routinely carried out by the outpatient service or telephone once every two months for the first two years after surgery and once every third month thereafter. Full physical examination, serological examination, and CT scan were conducted to determine recurrence. Serological examinations included liver function, AFP level, and a hepatitis virus test. If the patients were diagnosed with HCC recurrence [47], surgery, TACE, and other therapies including conservative therapy would be considered depending on the interval between last treatment and new diagnosis. The OS was calculated from the date of surgery to the date of the last follow-up or patient death. RFS was defined as the duration from the surgery date to the date of the first diagnosed recurrence or last follow-up [47].

### Statistical analysis

Student’s t-test or the Wilcoxon rank-sum test were used to compare continuous variables while the chi-square or Fisher’s exact test were used for categorical variables, using SPSS software, version 25 (IBM Corp., Armonk, NY). Independent risk factors associated with OS and RFS were evaluated in each group by using Cox regression analysis. OS and RFS curves based on sex difference were depicted through the Kaplan-Meier method. Nomograms integrated the results of the Cox regression multivariate analysis and were formulated by the package of rms in R version 3.5.1. The accuracy of prognostic prediction of the models increased with the value of the C-index. Calibration curves and decision curve analysis (DCA) were used to assess the performance of models including nomograms, according to methods detailed in our previous study [47]. Derived from two-tailed tests, P-values of <0.05 were defined as statistically significant. In observational studies, PSM has attracted increasing interest to avoid selection bias and increase the level of evidence [49]. To draw more reliable and confident results, PSM was performed at a ratio of 1:2 by the package of MatchIt in R version 3.5.1, using all potential covariates that could affect the group allocation [50].

## Supplementary Material

Supplementary Figures

Supplementary Tables
