# Supplementary material for: Effect of menopausal status on the survival and recurrence of sex-classified hepatocellular carcinoma after liver resection: a case-matched study with propensity score matching
Source: Aging (Albany NY). 2020 Nov 24;12(24):25895–915. doi: 10.18632/aging.202155 (PMC7803575; doi:10.18632/aging.202155)
Supplement: Supplementary Figures [file aging-12-202155-s001.pdf]

SUPPLEMENTARY FIGURES

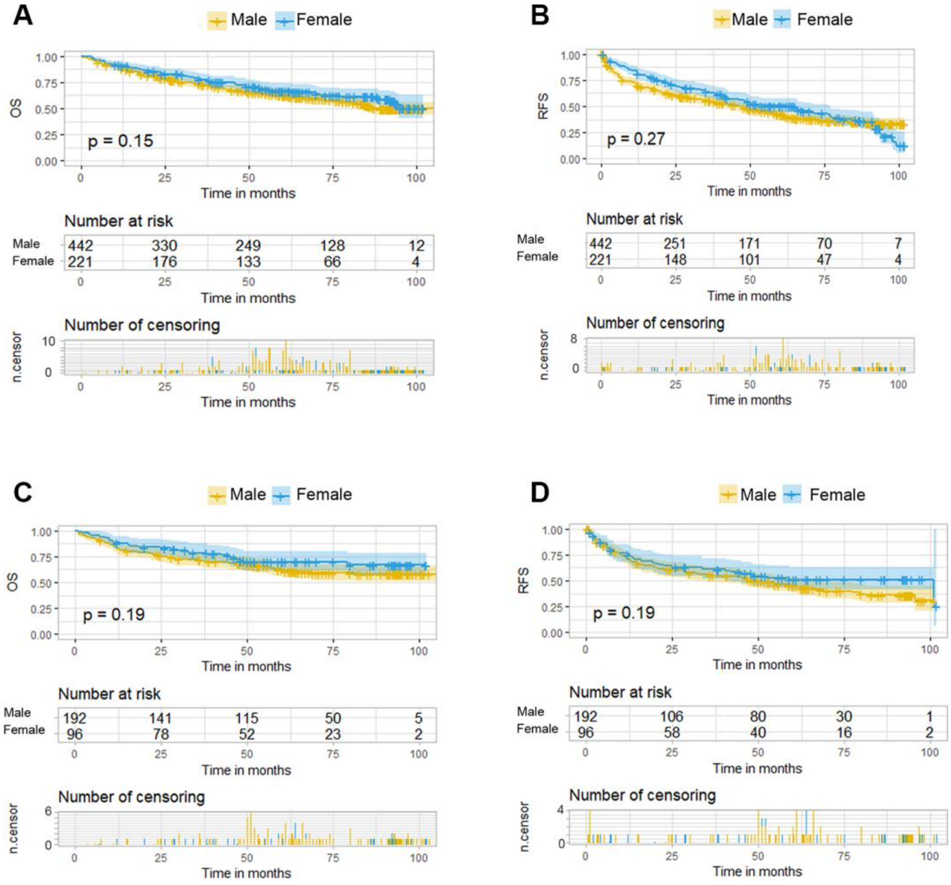

Supplementary Figure 1. Kaplan-Meier curves for overall survival (OS) and recurrence-free survival (RFS) of matched HCC patients grouped by sex after PSM. (**A**, **B**) KM curves of OS and RFS in the postmenopausal group matched with male patients. (**C**, **D**) KM curves of OS and RFS in the intermediate group matched with male patients.

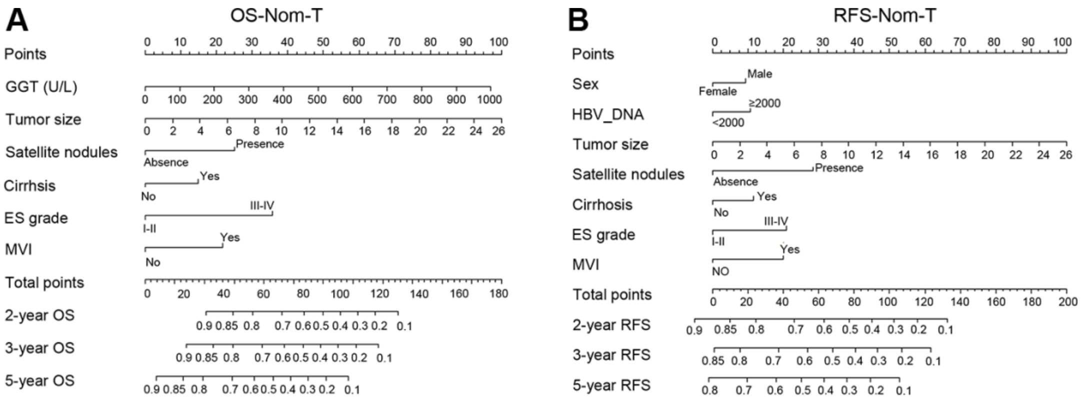

Supplementary Figure 2. Nomograms for predicting prognosis in the total patients with HCC. (**A**) nomogram model of overall survival (OS) (OS-Nom-T). (**B**) nomogram model of recurrence-free survival (RFS) (RFS-Nom-T).
