# Supplementary material for: Effect of menopausal status on the survival and recurrence of sex-classified hepatocellular carcinoma after liver resection: a case-matched study with propensity score matching
Source: Aging (Albany NY). 2020 Nov 24;12(24):25895–915. doi: 10.18632/aging.202155 (PMC7803575; doi:10.18632/aging.202155)
Supplement: Supplementary Tables [file aging-12-202155-s002.pdf]

SUPPLEMENTARY TABLES

Supplementary Table 1. Clinicopathological characteristics of all included HCC patients stratified by sex.

Features	Male (n=780)	Female (n=390)	*P*-value
Age (mean,Years)	53.87(10.17)	54.25(11.04)	0.555
Cigarette smoking (Yes/No)(%)	151(19.4)/629(80.6)	8(2.1)/382(97.9)	**<0.001**
Alcohol consumption (Yes/No)(%)	244(31.3)/536(68.7)	8(2.1)/382(97.9)	**<0.001**
Diabetes mellitus (Yes/No)(%)	169(21.7)/611(78.3)	47(12.1)/343(87.9)	**<0.001**
Antiviral (Yes/No)(%)	164(21.0)/616(79.0)	76(19.5)/314(80.5)	0.591
Tbi(mean, μmol/L)	14.51(9.78)	14.51(24.13)	0.999
Alb(mean, g/L)	41.25(3.92)	41.49(4.23)	0.335
ALT(mean, U/L)	36.74(28.83)	34.74 (33.54)	0.291
AST(mean, U/L)	36.71(28.37)	40.27(31.11)	0.051
GGT(mean, U/L)	82.28(83.56)	60.27(69.64)	**<0.001**
ALP(mean, U/L)	90.90(45.83)	96.48(49.88)	0.057
AFP(mean, ng/ml)	471.09(1973.27)	518.95(1039.32)	0.654
HBsAg(Positive/Negative)(%)	695(89.1)/85(10.9)	340(87.2)/50 (12.8)	0.382
HBV_DNA(≥2000/<2000 IU/ml)(%)	370(47.4)/410(52.6)	178(45.6)/212 (54.4)	0.605
Type of resection (Nonanatomical/anatomical)(%)	413(52.9)/367 (47.1)	200(51.3)/190 (48.7)	0.634
Surgical margin (<1/≥1cm)(%)	346(44.4)/434(55.6)	164(42.1)/226 (57.9)	0.492
Hilar clamping time (mean, minites)	15.38(8.54)	14.40(9.35)	0.071
Tumor size(mean, cm)	5.06(3.40)	4.76(3.28)	0.139
Tumor Number (>1/1)(%)	77(9.9)/703 (90.1)	25(6.4)/365(93.6)	0.062
Satellite nodules (presence/absence)(%)	96(12.3)/684 (87.7)	44(11.3)/346 (88.7)	0.679
Cirrhosis(Yes/No)(%)	499(64.0)/281(36.0)	253(64.9)/137(35.1)	0.812
Edmondson-Steiner grade III-IV/I-II(%)	525(67.3)/255(32.7)	257(65.9) /133(34.1)	0.677
MVI (Yes/No)(%)	87(11.2)/693(88.8)	60(15.4)/330(84.6)	**0.049**
BCLC stage (A-B/0)(%)	754(96.7/26(3.3)	374(95.9)/16(4.1)	0.617

Bold values indicate statistical significance ($P < 0.05$). *P* values male versus female cohort.
HCC, hepatocellular carcinoma; Tbi, total bilirubin; Alb, albumin; ALT, alanine transaminase; AST, aspartate aminotransferase; GGT, γ-glutamyl transferase; ALP, alkaline phosphatase; AFP indicates α-fetoprotein; MVI, microvascular invasion; BCLC, Barcelona Clinic Liver Cancer.

Supplementary Table 2. Univariate and multivariate analysis of OS and RFS in all HCC patients.

Features	OS			RFS		
	HR	95%CI	*P*-value	HR	95%CI	*P*-value
**Univariate analysis**						
Sex(Male/Female)	0.750	0.61-0.93	**0.007**	0.800	0.68-0.95	**0.010**
Age (mean,Years)	1.000	0.99-1.01	0.455	1.000	0.99-1.01	0.727
Cigarette smoking (Yes/No)(%)	1.170	0.91-1.51	0.216	1.040	0.84-1.3	0.699
Alcohol consumption (Yes/No)(%)	1.040	0.84-1.3	0.725	1.010	0.84-1.22	0.921
Diabetes mellitus (Yes/No)(%)	0.950	0.75-1.21	0.694	1.150	0.95-1.4	0.161
Antiviral (Yes/No)(%)	0.800	0.63-1.02	0.072	0.820	0.67-1.01	0.057
Tbi(mean, μmol/L)	1.010	1-1.01	**<0.001**	1.000	0.99-1.01	0.921
Alb(mean, g/L)	0.950	0.93-0.97	**<0.001**	0.950	0.94-0.97	**<0.001**
ALT(mean, U/L)	1.000	1-1.01	**0.030**	1.000	1-1.00	0.063
AST(mean, U/L)	1.000	1-1.01	**<0.001**	1.000	1-1.01	**<0.001**
GGT(mean, U/L)	1.000	1-1.00	**<0.001**	1.000	1-1.00	**<0.001**
ALP(mean, U/L)	1.010	1-1.01	**<0.001**	1.000	1-1.01	**<0.001**
AFP(mean, ng/ml)	1.000	1-1.00	**0.001**	1.000	1-1.00	0.165
HBsAg(Positive/Negative)(%)	0.980	0.74-1.3	0.886	1.160	0.91-1.49	0.234
HBV_DNA(≥2000/<2000 IU/ml)(%)	1.340	1.11-1.61	**0.002**	1.360	1.16-1.58	**<0.001**
Type of resection (Nonanatomical/anatomical)(%)	1.230	1.02-1.48	**0.029**	1.080	0.93-1.26	0.330
Surgical margin (<1/≥1cm)(%)	1.020	0.84-1.22	0.870	1.090	0.94-1.27	0.266
Hilar clamping time (mean, minites)	1.010	1-1.02	0.181	1.010	1-1.02	**0.014**
Tumor size(mean, cm)	1.110	1.09-1.14	**<0.001**	1.090	1.07-1.11	**<0.001**
Tumor Number (>1/1)(%)	1.530	1.14-2.06	**0.004**	1.660	1.29-2.14	**<0.001**
Satellite nodules (presence/absence)(%)	1.930	1.5-2.49	**<0.001**	1.970	1.58-2.46	**<0.001**
Cirrhosis(Yes/No)(%)	1.380	1.13-1.68	**0.002**	1.280	1.09-1.51	**0.003**
Edmondson-Steiner grade III-IV/I-II(%)	2.550	2.04-3.19	**<0.001**	1.660	1.4-1.96	**<0.001**
MVI (Yes/No)(%)	2.040	1.6-2.6	**<0.001**	1.790	1.44-2.22	**<0.001**
**Multivariate analysis**						
Sex(Male/Female)				0.810	0.68-0.96	**0.016**
HBV_DNA(≥2000/<2000 IU/ml)(%)				1.220	1.04-1.44	**0.013**
Tumor size(mean, cm)	1.090	1.06-1.12	**<0.001**	1.070	1.04-1.09	**<0.001**
Satellite nodules (presence/absence)(%)	2.230	1.36-3.67	**0.002**	2.260	1.47-3.48	**<0.001**
Cirrhosis(Yes/No)(%)	1.400	1.13-1.72	**0.002**	1.260	1.07-1.5	**0.007**
Edmondson-Steiner grade III-IV/I-II(%)	2.330	1.86-2.93	**<0.001**	1.550	1.3-1.84	**<0.001**
MVI (Yes/No)(%)	1.500	1.12-2.01	**0.006**	1.380	1.08-1.76	**0.009**

Bold values indicate statistical significance ($P < 0.05$).

HCC, hepatocellular carcinoma; Tbi, total bilirubin; Alb, albumin; ALT, alanine transaminase; AST, aspartate aminotransferase; GGT, γ-glutamyl transferase; ALP, alkaline phosphatase; AFP indicates α-fetoprotein; MVI, microvascular invasion; BCLC, Barcelona Clinic Liver Cancer; OS, overall survival; RFS, recurrence-free survival; CI, confidence interval; HR, hazard ratio.

Supplementary Table 3. Comparison of clinicopathological characteristics between postmenopausal females and their matched male patients.

Features	Male (n=442)	Female (n=221)	*P*-value
Age (mean,Years)	61.09(5.55)	62.09 (5.81)	**0.032**
Cigarette smoking (Yes/No)(%)	47(10.6)/395(89.4)	4(1.8)/217(98.2)	**<0.001**
Alcohol consumption (Yes/No)(%)	120 (27.1)/322 (72.9)	6(2.7)/215 (97.3)	**<0.001**
Diabetes mellitus (Yes/No)(%)	156(35.3)/286(64.7)	31(14.0)/190(86.0)	**<0.001**
Antiviral (Yes/No)(%)	84(19.0)/358 (81.0)	43(19.5)/178 (80.5)	0.972
Tbi(mean, μmol/L)	15.33(10.35)	15.77(31.67)	0.789
Alb(mean, g/L)	41.11(3.92)	41.22(4.33)	0.757
ALT(mean, U/L)	34.07(23.63)	33.08(23.44)	0.609
AST(mean, U/L)	35.96(30.03)	39.89(24.29)	0.092
GGT(mean, U/L)	79.58(83.38)	51.23(40.54)	**<0.001**
ALP(mean, U/L)	91.75(48.34)	100.30(42.44)	**0.026**
AFP(mean, ng/ml)	427.58(2199.92)	427.67 (947.55)	1.000
HBsAg(Positive/Negative)(%)	384(86.9)/58(13.1)	187(84.6)/34(15.4)	0.500
HBV_DNA(≥2000/<2000 IU/ml)(%)	212(48.0)/230(52.0)	98(44.3)/123 (55.7)	0.425
Type of resection (Nonanatomical/anatomical)(%)	237(53.6)/205(46.4)	111(50.2)/110 (49.8)	0.458
Surgical margin (<1/≥1cm)(%)	206(46.6)/236(53.4)	89(40.3)/132(59.7)	0.143
Hilar clamping time (mean, minites)	14.66(7.89)	13.34(7.49)	**0.040**
Tumor size(mean, cm)	4.77(3.06)	4.33(2.51)	0.066
Tumor Number (>1/1)(%)	50(11.3)/392(88.7)	15(6.8)/206(93.2)	0.088
Satellite nodules (presence/absence)(%)	50(11.3)/392(88.7)	15(6.8)/206(93.2)	0.088
Cirrhosis(Yes/No)(%)	270(61.1)/172(38.9)	155(70.1)/66(29.9)	**0.028**
Edmondson-Steiner grade III-IV/I-II(%)	284(64.3)/158(35.7)	154(69.7)/67 (30.3)	0.192
MVI (Yes/No)(%)	40(9.0)/402(91.0)	32(14.5)/189 (85.5)	**0.047**
BCLC stage (A-B/0)(%)	429(97.1)/13(2.9)	215(97.3)/6(2.7)	1.000

Bold values indicate statistical significance ($P < 0.05$). P values male versus female cohort.
HCC, hepatocellular carcinoma; Tbi, total bilirubin; Alb, albumin; ALT, alanine transaminase; AST, aspartate aminotransferase; GGT, γ-glutamyl transferase; ALP, alkaline phosphatase; AFP indicates α-fetoprotein; MVI, microvascular invasion; BCLC, Barcelona Clinic Liver Cancer.

Supplementary Table 4. Univariate and multivariate analysis of OS and RFS in the postmenopausal group matched with male HCC patients after PSM.

Features	OS			RFS		
	HR	95%CI	*P*-value	HR	95%CI	*P*-value
**Univariate analysis**						
Sex(Male/Female)	0.830	0.63-1.08	0.155	0.890	0.72-1.1	0.266
Age (mean,Years)	1.010	0.99-1.03	0.484	0.980	0.97-1	0.094
Cigarette smoking (Yes/No)(%)	1.040	0.67-1.63	0.861	1.140	0.8-1.63	0.481
Alcohol consumption (Yes/No)(%)	0.790	0.57-1.1	0.164	0.860	0.66-1.12	0.262
Diabetes mellitus (Yes/No)(%)	1.040	0.8-1.37	0.760	1.100	0.88-1.37	0.421
Antiviral (Yes/No)(%)	0.720	0.51-1.02	0.066	0.900	0.69-1.18	0.451
Tbi(mean, μmol/L)	1.010	1.01-1.02	**<0.001**	1.000	0.99-1.02	0.589
Alb(mean, g/L)	0.930	0.91-0.96	**<0.001**	0.950	0.93-0.97	**<0.001**
ALT(mean, U/L)	1.000	1-1.01	0.071	1.000	1-1.01	**0.049**
AST(mean, U/L)	1.000	1-1.01	**0.001**	1.000	1-1.01	**0.003**
GGT(mean, U/L)	1.000	1-1.00	**<0.001**	1.000	1-1.00	**<0.001**
ALP(mean, U/L)	1.010	1-1.01	**<0.001**	1.000	1-1.01	**<0.001**
AFP(mean, ng/ml)	1.000	1-1.00	**0.002**	1.000	1-1.00	0.063
HBsAg(Positive/Negative)(%)	0.790	0.57-1.1	0.169	1.060	0.79-1.42	0.717
HBV_DNA(≥2000/<2000 IU/ml)(%)	1.160	0.91-1.48	0.222	1.310	1.08-1.6	**0.007**
Type of resection (Nonanatomical/anatomical)(%)	1.270	0.99-1.62	0.059	1.080	0.88-1.31	0.477
Surgical margin (<1/≥1cm)(%)	0.930	0.73-1.19	0.571	1.010	0.82-1.23	0.953
Hilar clamping time (mean, minites)	1.000	0.99-1.02	0.674	1.010	0.99-1.02	0.263
Tumor size(mean, cm)	1.130	1.09-1.17	**<0.001**	1.090	1.06-1.13	**<0.001**
Tumor Number (>1/1)(%)	1.650	1.15-2.38	**0.007**	1.600	1.16-2.22	**0.004**
Satellite nodules (presence/absence)(%)	1.650	1.15-2.38	**0.007**	1.600	1.16-2.22	**0.004**
Cirrhosis(Yes/No)(%)	1.710	1.3-2.25	**<0.001**	1.490	1.2-1.84	**<0.001**
Edmondson-Steiner grade III-IV/I-II(%)	2.690	1.99-3.62	**<0.001**	1.650	1.33-2.06	**<0.001**
MVI (Yes/No)(%)	1.690	1.19-2.38	**0.003**	1.730	1.29-2.32	**<0.001**
**Multivariate analysis**						
Tbi(mean, μmol/L)	1.010	1-1.01	**0.001**			
Alb(mean, g/L)	0.960	0.93-1	**0.032**			
Tumor size(mean, cm)	1.160	1.11-1.21	**<0.001**	1.100	1.06-1.14	**<0.001**
Tumor Number (>1/1)(%)	1.750	1.2-2.55	**0.004**	1.530	1.09-2.14	**0.014**
Cirrhosis(Yes/No)(%)	1.860	1.39-2.5	**<0.001**	1.430	1.14-1.79	**0.002**
Edmondson-Steiner grade III-IV/I-II(%)	2.630	1.94-3.56	**<0.001**	1.640	1.31-2.05	**<0.001**

Bold values indicate statistical significance ($P < 0.05$).
HCC, hepatocellular carcinoma; Tbi, total bilirubin; Alb, albumin; ALT, alanine transaminase; AST, aspartate aminotransferase; GGT, γ-glutamyl transferase; ALP, alkaline phosphatase; AFP indicates α-fetoprotein; MVI, microvascular invasion; BCLC, Barcelona Clinic Liver Cancer; OS, overall survival; RFS, recurrence-free survival; CI, confidence interval; HR, hazard ratio.

Supplementary Table 5. Comparison of clinicopathological characteristics between intermediate group and matched male patients with HCC after PSM.

Features	Male (n=192)	Female (n=96)	*P*-value
Age (mean,Years)	48.94 (2.81)	48.95(2.91)	0.977
Cigarette smoking (Yes/No)(%)	29(15.1)/163 (84.9)	2(2.1)/94(97.9)	**0.002**
Alcohol consumption (Yes/No)(%)	41(21.4)/151(78.6)	0(0.0)/96(100.0)	**<0.001**
Diabetes mellitus (Yes/No)(%)	7(3.6)/185(96.4)	9(9.4)/87(90.6)	0.084
Antiviral (Yes/No)(%)	41(21.4)/151(78.6)	24(25.0)/72(75.0)	0.584
Tbi(mean, µmol/L)	14.17(7.84)	13.71(5.79)	0.612
Alb(mean, g/L)	40.94(4.03)	41.43(4.01)	0.324
ALT(mean, U/L)	43.25(34.74)	39.97(38.13)	0.466
AST(mean, U/L)	38.95(25.08)	39.66(25.43)	0.821
GGT(mean, U/L)	95.64(93.17)	82.39(103.12)	0.273
ALP(mean, U/L)	91.48(40.20)	91.98(41.06)	0.922
AFP(mean, ng/ml)	424.21(946.56)	609.74(1438.05)	0.192
HBsAg(Positive/Negative)(%)	167(87.0)/25(13.0)	85(88.5)/11(11.5)	0.850
HBVDNA(≥2000/<2000 IU/ml)(%)	85(44.3)/107(55.7)	45(46.9)/51(53.1)	0.769
Type of resection (Nonanatomical/anatomical)(%)	103(53.6)/89(46.4)	56(58.3)/40(41.7)	0.530
Surgical margin (<1/≥1cm)(%)	80 (41.7) / 112(58.3)	56(58.3)/40(41.7)	0.705
Hilar clamping time (mean, minites)	16.71(10.06)	14.88(10.16)	0.147
Tumor size(mean, cm)	5.60(3.76)	4.66(3.15)	**0.036**
Tumor Number (>1/1)(%)	14(7.3)/178(92.7)	4(4.2)/92 (95.8)	0.439
Satellite nodules (presence/absence)(%)	15(7.8)/177(92.2)	4(4.2)/92(95.8)	0.356
Cirrhosis(Yes/No)(%)	128(66.7)/64(33.3)	56(58.3)/40(41.7)	0.208
Edmondson-Steiner grade III-IV/I-II(%)	138(71.9)/54(28.1)	65(67.7)/31(32.3)	0.553
MVI (Yes/No)(%)	14(7.3)/178(92.7)	2(2.1)/94(97.9)	0.122
BCLC stage (A-B/0)(%)	190(99.0)/2(1.0)	88(91.7)/8(8.3)	**0.004**

Bold values indicate statistical significance ($P < 0.05$). *P* values male versus female cohort.
HCC, hepatocellular carcinoma; Tbi, total bilirubin; Alb, albumin; ALT, alanine transaminase; AST, aspartate aminotransferase; GGT, γ-glutamyl transferase; ALP, alkaline phosphatase; AFP indicates α-fetoprotein; MVI, microvascular invasion; BCLC, Barcelona Clinic Liver Cancer.

Supplementary Table 6. Univariate and multivariate analysis of OS and RFS in the intermediate group matched male patients with HCC after PSM.

Features	OS			RFS		
	HR	95%CI	*P*-value	HR	95%CI	*P*-value
**Univariate analysis**						
Sex	0.750	0.48-1.15	0.187	0.790	0.56-1.12	0.188
Age (mean,Years)	0.960	0.9-1.03	0.248	1.010	0.95-1.07	0.825
Cigarette smoking (Yes/No)(%)	1.200	0.66-2.2	0.551	0.950	0.57-1.61	0.862
Alcohol consumption (Yes/No)(%)	1.300	0.77-2.19	0.323	1.060	0.68-1.66	0.785
Diabetes mellitus (Yes/No)(%)	0.290	0.07-1.17	0.082	1.080	0.55-2.13	0.813
Antiviral (Yes/No)(%)	0.650	0.39-1.1	0.109	0.770	0.52-1.15	0.203
Tbi(mean, µmol/L)	1.010	1-1.03	0.103	1.010	0.98-1.03	0.554
Alb(mean, g/L)	0.950	0.9-1	**0.039**	0.960	0.92-1	**0.033**
ALT(mean, U/L)	1.010	1-1.01	**<0.001**	1.000	1-1.01	**0.026**
AST(mean, U/L)	1.010	1.01-1.02	**<0.001**	1.010	1.01-1.02	**<0.001**
GGT(mean, U/L)	1.000	1-1.01	**<0.001**	1.000	1-1.00	**0.005**
ALP(mean, U/L)	1.010	1-1.01	**<0.001**	1.010	1-1.01	**<0.001**
AFP(mean, ng/ml)	1.000	1-1.00	0.432	1.000	1-1.00	0.179
HBsAg(Positive/Negative)(%)	1.450	0.75-2.79	0.264	1.280	0.78-2.1	0.330
HBVDNA(≥2000/<2000 IU/ml)(%)	1.680	1.13-2.49	**0.010**	1.360	0.99-1.87	0.060
Type of resection (Nonanatomical/anatomical)(%)	1.380	0.92-2.06	0.116	1.200	0.87-1.66	0.273
Surgical margin (<1/≥1cm)(%)	0.780	0.53-1.17	0.235	1.210	0.88-1.68	0.236
Hilar clamping time (mean, minites)	1.020	1-1.03	0.066	1.020	1-1.03	**0.042**
Tumor size(mean, cm)	1.120	1.07-1.16	**<0.001**	1.090	1.05-1.14	**<0.001**
Tumor Number (>1/1)(%)	2.360	1.23-4.55	**0.010**	2.030	1.14-3.59	**0.015**
Satellite nodules (presence/absence)(%)	2.110	1.1-4.06	**0.025**	1.940	1.12-3.38	**0.018**
Cirrhosis(Yes/No)(%)	0.930	0.62-1.39	0.725	0.990	0.71-1.39	0.970
Edmondson-Steiner grade III-IV/I-II(%)	3.610	2.01-6.46	**<0.001**	1.510	1.05-2.16	**0.025**
MVI (Yes/No)(%)	2.570	1.37-4.82	**0.003**	1.170	0.57-2.38	0.674
**Multivariate analysis**						
AST(mean, U/L)				1.010	1-1.02	**0.020**
GGT(mean, U/L)	1.000	1-1.01	**0.008**			
Edmondson-Steiner grade III-IV/I-II(%)	2.530	1.38-4.62	**0.003**			
Tumor size(mean, cm)	1.100	1.04-1.16	**<0.001**	1.060	1.01-1.11	**0.016**

Bold values indicate statistical significance ($P < 0.05$).
HCC, hepatocellular carcinoma; Tbi, total bilirubin; Alb, albumin; ALT, alanine transaminase; AST, aspartate aminotransferase; GGT, γ-glutamyl transferase; ALP, alkaline phosphatase; AFP indicates α-fetoprotein; MVI, microvascular invasion; BCLC, Barcelona Clinic Liver Cancer; OS, overall survival; RFS, recurrence-free survival; CI, confidence interval; HR, hazard ratio.
